# A new integrative analysis of histopathology and single cell RNA-seq reveals the CCL5 mediated T and NK cell interaction with vascular cells in idiopathic pulmonary arterial hypertension

**DOI:** 10.1186/s12967-024-05304-6

**Published:** 2024-05-26

**Authors:** Xincheng Li, Shuangshuang Ma, Qi Wang, Yishan Li, Xiaofan Ji, Jixiang Liu, Jing Ma, Yongbing Wang, Zhu Zhang, Hong Zhang, Hong Chen, Linfeng Xi, Yunxia Zhang, Wanmu Xie, Lu Sun, Zhihui Fu, Peiran Yang, Chen Wang, Zhenguo Zhai

**Affiliations:** 1 Department of Respiratory and Critical Care Medicine, The Second Affiliated Hospital of Harbin Medical University, Harbin, 150081 China; 2 National Center for Respiratory Medicine; State Key Laboratory of Respiratory Health and Multimorbidity; National Clinical Research Center for Respiratory Diseases; Institute of Respiratory Medicine, Chinese Academy of Medical Sciences; Department of Pulmonary and Critical Care Medicine, Center of Respiratory Medicine, China-Japan Friendship Hospital, Beijing, 100029 China; 3 Beijing University of Chinese Medicine, Beijing, 100029 China; 4 The First Clinical Medical College, Shanxi Medical University, Taiyuan, 030001 China; 5 State Key Laboratory of Respiratory Health and Multimorbidity, Department of Physiology, Institute of Basic Medical Sciences, Chinese Academy of Medical Sciences and School of Basic Medicine, Peking Union Medical College; National Center for Respiratory Medicine; Institute of Respiratory Medicine, Chinese Academy of Medical Sciences; National Clinical Research Center for Respiratory Diseases, Beijing, 100730 China

**Keywords:** IPAH, Inflammation, Single-cell RNA-sequencing, Cell-cell interaction

## Abstract

**Background:**

Inflammation and dysregulated immunity play vital roles in idiopathic pulmonary arterial hypertension (IPAH), while the mechanisms that initiate and promote these processes are unclear.

**Methods:**

Transcriptomic data of lung tissues from IPAH patients and controls were obtained from the Gene Expression Omnibus database. Weighted gene co-expression network analysis (WGCNA), differential expression analysis, protein-protein interaction (PPI) and functional enrichment analysis were combined with a hemodynamically-related histopathological score to identify inflammation-associated hub genes in IPAH. The monocrotaline-induced rat model of pulmonary hypertension was utilized to confirm the expression pattern of these hub genes. Single-cell RNA-sequencing (scRNA-seq) data were used to identify the hub gene-expressing cell types and their intercellular interactions.

**Results:**

Through an extensive bioinformatics analysis, CXCL9, CCL5, GZMA and GZMK were identified as hub genes that distinguished IPAH patients from controls. Among these genes, pulmonary expression levels of Cxcl9, Ccl5 and Gzma were elevated in monocrotaline-exposed rats. Further investigation revealed that only CCL5 and GZMA were highly expressed in T and NK cells, where CCL5 mediated T and NK cell interaction with endothelial cells, smooth muscle cells, and fibroblasts through multiple receptors.

**Conclusions:**

Our study identified a new inflammatory pathway in IPAH, where T and NK cells drove heightened inflammation predominantly via the upregulation of CCL5, providing groundwork for the development of targeted therapeutics.

**Supplementary Information:**

The online version contains supplementary material available at 10.1186/s12967-024-05304-6.

## Introduction

Idiopathic pulmonary arterial hypertension (IPAH) is a devastating pulmonary vascular disease characterized by a progressive increase in pulmonary vascular resistance and right heart failure [1]. Pulmonary vascular remodeling, marked by arteriolar eccentricity and occlusive intimal thickening, is central to the irreversible progression of IPAH [2]. Various drugs have been developed for the treatment of IPAH, including phosphodiesterase-5 inhibitors and soluble guanylate cyclase stimulators, endothelin receptor antagonists, prostacyclin analogues and prostacyclin I2 receptor agonists [3]. These drugs primarily alleviate symptoms by dilating pulmonary vessels, rather than effectively inhibiting the vascular remodeling, which is the core pathobiological process underlying the development of IPAH [1]. Consequently, improving the prognosis of patients with IPAH still remains a major challenge [4, 5]. It is therefore essential to undertake further research to elucidate the mechanisms of pulmonary vascular remodeling and identify novel therapeutic targets.

Growing evidence suggests that hyperactive inflammation and immune system dysregulation play a role in the pathobiology of IPAH by promoting vascular remodeling [2, 6–9], though the specific underlying mechanism remains elusive. Accumulation of immune cells in remodeled pulmonary arterioles is a common finding in IPAH lung tissue [2, 6], and the structural remodeling process overlaps with ongoing vascular inflammation [2]. Compelling evidence indicates that the vascular inflammation and an imbalanced immune response cause endothelial cell dysfunction [7] and abnormal proliferation of pulmonary vascular smooth muscle cells [8], ultimately resulting in pulmonary vascular remodeling [9]. Despite the clear link between immune system dysregulation and IPAH, it has proven difficult to quantify the extent of pulmonary vascular immune cell infiltration. Recently, a quantitative parameter, designated as an inflammatory score, has been developed for assessing the perivascular infiltration of immune cells in IPAH on the basis of histopathology, with higher scores indicating more severe inflammatory damage [2]. Leveraging this score, it may be possible to identify the principal genes and immune cell types driving this inflammatory process and confirm their role as key mediators of pathological vascular remodeling.

As sequencing technologies advance, the number of transcriptomic studies on pulmonary vascular diseases has expanded rapidly, encompassing both microarray [10, 11] and single cell RNA sequencing (scRNA-seq) studies [12]. Despite the vast amount of data available, effectively integrating these data to identify key genes triggering the pathobiological vascular remodeling remains a formidable challenge. It is important to recognize that microarray data lack cell type-specific resolution, while scRNA-seq data are often obtained from a limited number of samples. The combination of these approaches may provide a more complete transcriptomic landscape and the implicated cell types, but the lack of histological information on the lung tissue could obscure the local pathobiological status, resulting in misinterpretation of the transcriptomic data [13]. As previously mentioned, the inflammatory score acts as a direct histopathological marker that reflects the perivascular inflammatory response. By incorporating this score, it is possible to identify genes closely associated with pulmonary arterial inflammation from a large set of transcripts [11]. This discovery suggests that integrating the inflammatory score can facilitate the interpretation of transcriptomic data based on the actual histopathological states, thereby enabling a comprehensive analysis of the molecular mechanisms of immune cell infiltration and its impact on vascular remodeling in IPAH.

In this study, we combined transcriptomic data with a clinically relevant inflammatory score and experimental validation, in order to identify critical genes associated with inflammation and decipher their cellular regulatory networks, thereby providing new insights into the inflammatory mechanisms of IPAH.

## Materials and methods

### Dataset acquisition and preprocessing

In this study, the microarray sequencing dataset GSE117261 and the scRNA-seq dataset GSE169471 were retrieved from the GEO database. Information related to these two datasets was summarized in Additional file 1: Table S1. The methodological details for evaluating the pulmonary arterial inflammatory score in this dataset are thoroughly documented elsewhere [2]. In brief, this score reflects the density and distribution of perivascular inflammatory cells, assigned based on histological examinations of tissue sections as follows: 0 for no perivascular infiltration, 1 for minimal infiltration, 2 for moderate localized aggregation, and 3 for extensive infiltration, where large clusters extend beyond the perivascular area into adjacent alveoli. The score is calculated by summing the products of each score (0, 1, 2, or 3) and the corresponding number of vessels, then dividing by the total vessels analyzed. Scores range from 0.0 to 3.0, with higher values indicating more severe vascular inflammatory infiltration. After excluding participants under 18 years of age (*n* = 9) and a participant with incomplete clinical data (*n* = 1), the analysis proceeded with 21 normal individuals and 26 IPAH patients.

### Weighted co-expression network construction

The WGCNA software package (version 1.71) was used to construct gene co-expression networks [14]. Initially, hierarchical clustering analysis was employed to identify and remove outliers. The soft-threshold power was determined using the “pickSoftThreshold” function. The adjacency matrix was then transformed into a topological overlap matrix with a minimum module size of 30 to identify the gene modules. Clinical data associated with the samples, including the inflammatory score, were integrated with WGCNA results to explore their relationship with the gene modules, facilitating the identification of a gene co-expression module closely associated with the inflammatory score.

### Differential expression analysis

Differential expression analysis was performed using the “limma” package (version 3.50.0), comparing IPAH patients with controls [15]. The “normalizeBetweenArrays” function was used to correct for potential technical errors and to minimize batch effects across 45 samples. Differentially expressed genes (DEGs) identified based on criteria of an absolute log2 fold-change greater than 0.58, which corresponds to a 1.5-fold change in expression between the groups, and an adjusted p-value of less than 0.05.

### Identification of inflammation-related DEGs

The list of DEGs was cross-referenced with the list of genes in the inflammatory score-associated co-expression module obtained from WGCNA. The intersecting genes were designated as inflammation-related DEGs and used for subsequent analyses.

### Functional enrichment analysis

The functional enrichment analysis of inflammation-related DEGs was conducted using Gene Ontology (GO) and the Kyoto Encyclopedia of Genes and Genomes (KEGG). The GO and KEGG analyses were performed using the “clusterProfiler” package (version 4.2.0) [16].

### Protein-protein interaction network construction and hub gene identification

The protein-protein interaction (PPI) network for the inflammation-related DEGs was constructed using the STRING database (https://cn.string-db.org/). Interaction pairs with a PPI combined score greater than 0.4 were deemed significant [17]. Then, the network data were imported into the Cytoscape software, where six algorithms (Degree, Closeness, Stress, EPC, MCC, and MNC) were employed to calculate the degree of connectivity of the inflammation-related DEGs [18]. Hub genes were determined as the overlapping top 5 genes across these algorithms.

### Construction of the monocrotaline-induced rat model of pulmonary hypertension

The protocol of the animal study was approved by the Medical Ethics Committee of the Second Affiliated Hospital of Harbin Medical University (YJSDW2022-090). Sprague Dawley rats (6-week-old, male) were randomly divided into two groups: the monocrotaline (MCT) group (*n* = 6) received a single subcutaneous injection of MCT (60 mg/kg, Sigma), whereas the control group (*n* = 6) received an equivalent volume of saline. After 21 days, the right ventricular systolic pressure was measured under isoflurane anesthesia using a Millar catheter (SPR-513) inserted into the right jugular vein. Subsequently, the animals were euthanized by exsanguination, and the lung and heart tissues were collected. The degree of right ventricular hypertrophy was calculated as the weight ratio between the right ventricle versus the left ventricle plus septum (RV/ (LV + S)). Pulmonary vascular morphology was assessed using Hematoxylin and Eosin staining.

### RNA extraction and real-time quantitative PCR

Total RNA was extracted from lung tissue samples and reverse transcribed into cDNA using the ReverTra Ace™ RT kit (TOYOBO). Gene expression was quantified using real-time quantitative PCR (RT-qPCR), using the 2-ΔΔCt method to determine the relative expression levels. Primer sequences can be found in the supplementary materials (Additional file 2: Table S2).

### ScRNA-seq data analysis

The scRNA-seq data (GSE169471) were processed using the “Seurat” package (version 4.0.5) [19]. Low-quality cells were filtered out based on the following criteria: detection of fewer than 200 unique genes or genes present in fewer than three cells; (2) presence of more than 3,000 feature RNAs; and (3) a mitochondrial gene content exceeding 10%. Principal component analysis (PCA) and uniform manifold approximation and projection (UMAP) analysis were used for dimension reduction and clustering. The “sctransform” package (version 0.3.2) was used to integrate multiple samples [19]. The “FindAllMarkers” function was utilized to identify marker genes for each cell cluster. Subsequently, the clusters were annotated using the CellMarker [20] and PanglaoDB databases [21], and the original publication associated with the dataset [12]. Cell-cell communications between the identified cell subsets and immune cells were deciphered by mapping receptor-ligand pairs with iTALK (version 0.1.0) [22].

### Statistical analysis

Statistical analyses were conducted using R software (version 4.1.1) and SPSS (version 26.0). Continuous variables were compared using the Wilcoxon rank-sum test or Student’s t-test, where appropriate. In order to assess the diagnostic performance of genes, the receiver operating characteristic (ROC) curve analysis was performed and the area under the curve (AUC) values were calculated. All tests were two-sided, and a *p*-value < 0.05 was considered statistically significant.

## Results

### Identification of a gene co-expression module associated with the inflammatory score

**Fig. 1:**
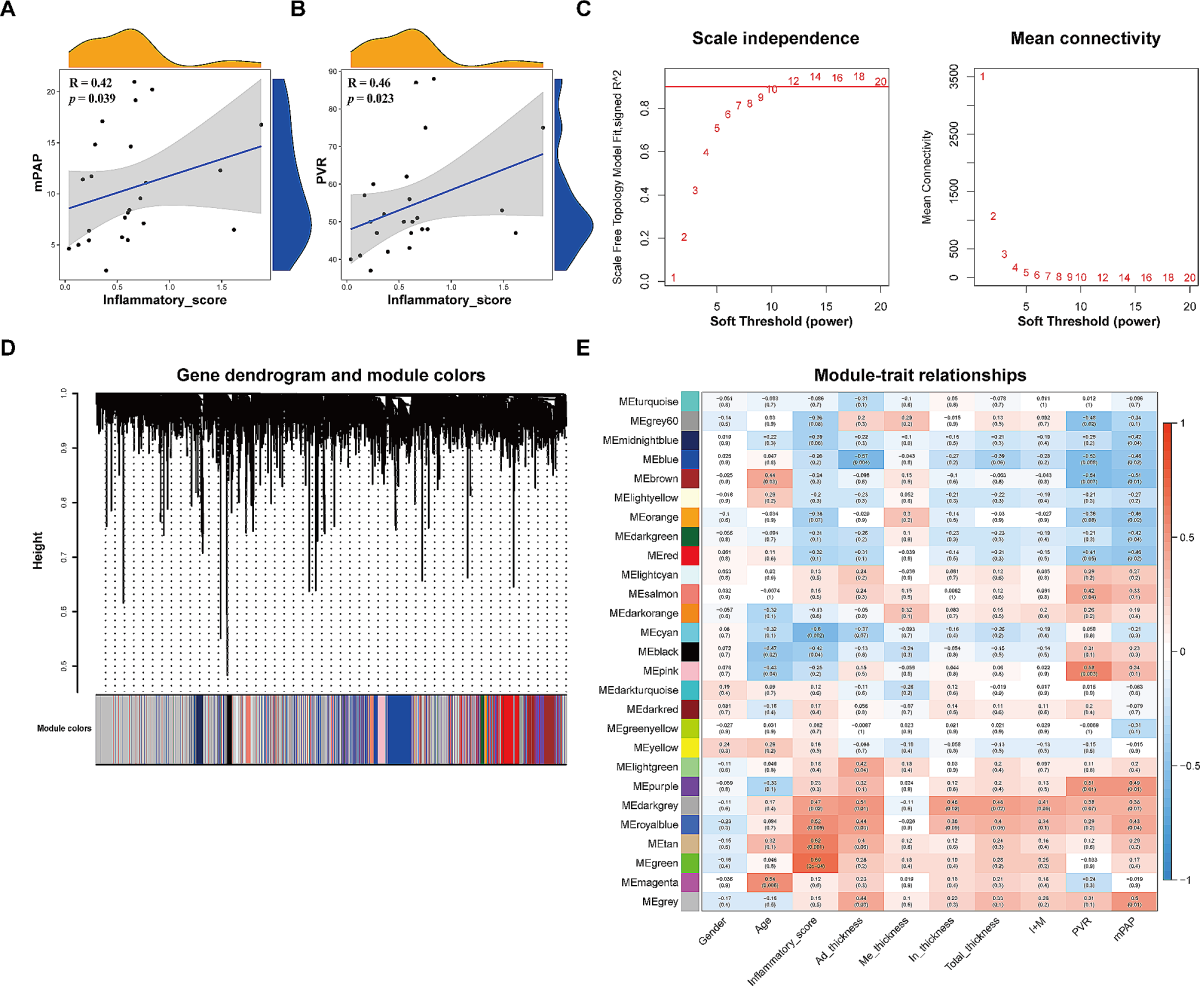
Identification of inflammatory score-associated genes through weighted gene co-expression network analysis (WGCNA). **A. **Spearman correlation scatterplot showing the relationship between the inflammatory score and mean pulmonary artery pressure (mPAP), with a positive correlation indicated (*R* = 0.42, *p* = 0.03). **B. **Spearman correlation scatterplot of the inflammatory score against pulmonary vascular resistance (PVR), also depicting a positive correlation (*R* = 0.46, *p* = 0.02). **C. **Analysis of network topology for various soft-thresholding powers to ensure a scale-free network; the left panel illustrates the scale-free fit index (y-axis) as a function of the soft-thresholding power (x-axis), while the right panel displays the mean connectivity (degree of gene co-expression) as a function of the soft-thresholding power. **D. **Dendrogram generated from the hierarchical clustering of gene modules identified by WGCNA, with module colors below the dendrogram indicating gene clustering. Grey modules signify genes that could not be clustered into any of the modules. **E.** Heatmap displaying module-trait relationships, with color intensity reflecting the degree of correlation (red for positive, blue for negative). Each row represents a gene module designated by color, and each column represents a clinical trait. The green gene module had the highest correlation with the inflammation score, with a correlation coefficient of 0.69

In order to integrate transcriptomic data with histopathology and clinical variables, WGCNA was performed to facilitate the identification of gene co-expression modules correlated with the inflammatory score. Initially, two outlier samples were removed based on hierarchical clustering analysis (Additional file 3, Fig.S1). Consequently, the study included 24 IPAH and 21 control samples with complete clinical data (Additional file 4, Table S3), with a median inflammatory score of 0.60 (0.26–0.74). Notably, spearman analysis showed that the inflammatory score exhibited a significantly positive correlation with mean pulmonary artery pressure (mPAP, *R* = 0.42, *p* = 0.039) and pulmonary vascular resistance (PVR, *R* = 0.46, *p* = 0.023) in IPAH patients (Fig. 1A, B), supporting its relevance to important clinical parameters. The soft threshold (power) was determined to be 10, based on the optimal scale-free fit index and mean connectivity (Fig. 1C), resulting in the identification of 27 gene co-expression modules (Fig. 1D). As shown in Fig. 1E, the green module, encompassing 665 genes, displayed a strong association with the inflammatory score based on Pearson’s correlation analysis (correlation coefficient = 0.69, *p* = 2e-04). Additionally, a significant correlation (correlation coefficient = 0.59, *p* = 1.3e-63) was also observed between the module membership (MM) and gene significance (GS) within this module (Additional file 5: Fig. S2). Taken together, a set of genes have been isolated from the transcriptomic data owing to their associations with the clinically relevant inflammatory score.

### Identification of inflammation-related DEGs in IPAH and their functions

**Fig. 2:**
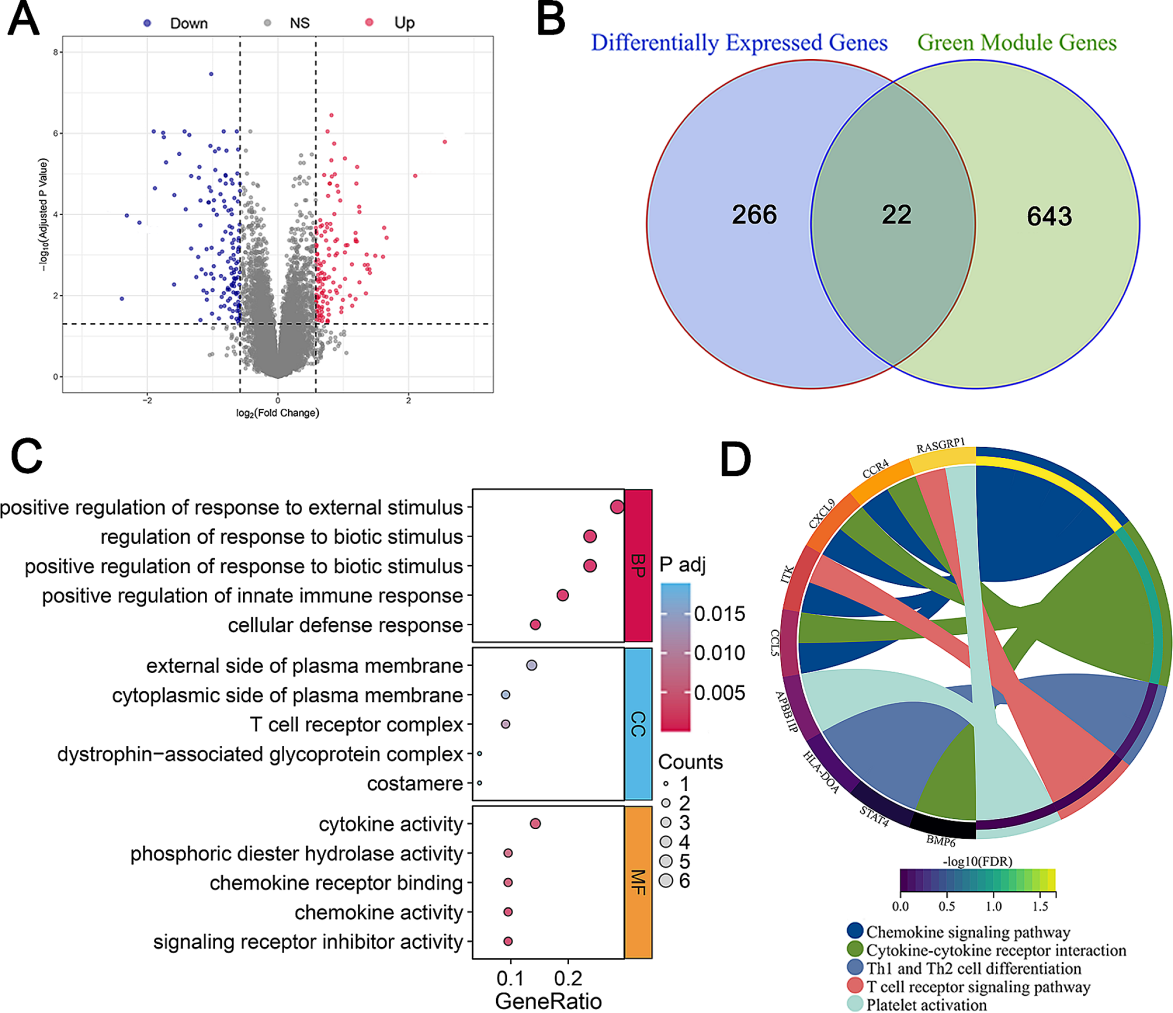
Comprehensive gene expression and enrichment analysis of idiopathic pulmonary arterial hypertension (IPAH). **A**. Volcano plot displaying differentially expressed genes (DEGs) between IPAH and normal samples. DEGs were represented as dots, with upregulated genes in red, downregulated genes in blue, and non-significant genes in gray. **B**. Venn diagram showing the overlap between DEGs and genes from the green co-expression module associated with the inflammatory score, highlighting 22 genes common to both datasets. **C**. Gene Ontology (GO) enrichment analysis of the 22 intersecting genes, categorizing them into biological processes (BP), cellular components (CC), and molecular functions (MF), with the size of the dots indicating the gene count and color gradient representing the adjusted *p*-value. **D**. Kyoto Encyclopedia of Genes and Genomes (KEGG) pathway analysis for the intersecting genes, where each colored band represents a pathway linked to the genes listed, with the width of the bands correlating to the -log10 of the false discovery rate (FDR) adjusted *p*-values, indicating the significance of the gene-pathway association

Given that potentially important genes are likely to display altered expression levels in diseased tissues, a differential expression analysis was carried out. The gene expression profiles of all 45 samples were subjected to normalization (Additional file 6: Fig.S3A, B). Comparing IPAH samples with normal controls yielded 288 DEGs (Additional file 7: Table S4), as presented in Fig. 2A. In order to isolate DEGs associated with the inflammatory score, the intersection of the 288 DEGs with the WGCNA-derived inflammatory score module resulted in 22 genes (Fig. 2B). GO function and KEGG pathway analyses were performed to delineate the potential roles of these 22 inflammation-related genes. As expected, GO analysis indicated significant enrichment in immune response and cytokine activity (Fig. 2C), while KEGG results showed significant enrichment in crucial pathways such as chemokine signaling, Th1 and Th2 cell differentiation, T cell receptor signaling, and platelet activation (Fig. 2D). Collectively, this series of analyses uncovered 22 genes that not only demonstrated significantly altered expression in disease but also showed a strong association with histopathological inflammation.

### Discovery of hub genes for vascular inflammation

**Fig. 3:**
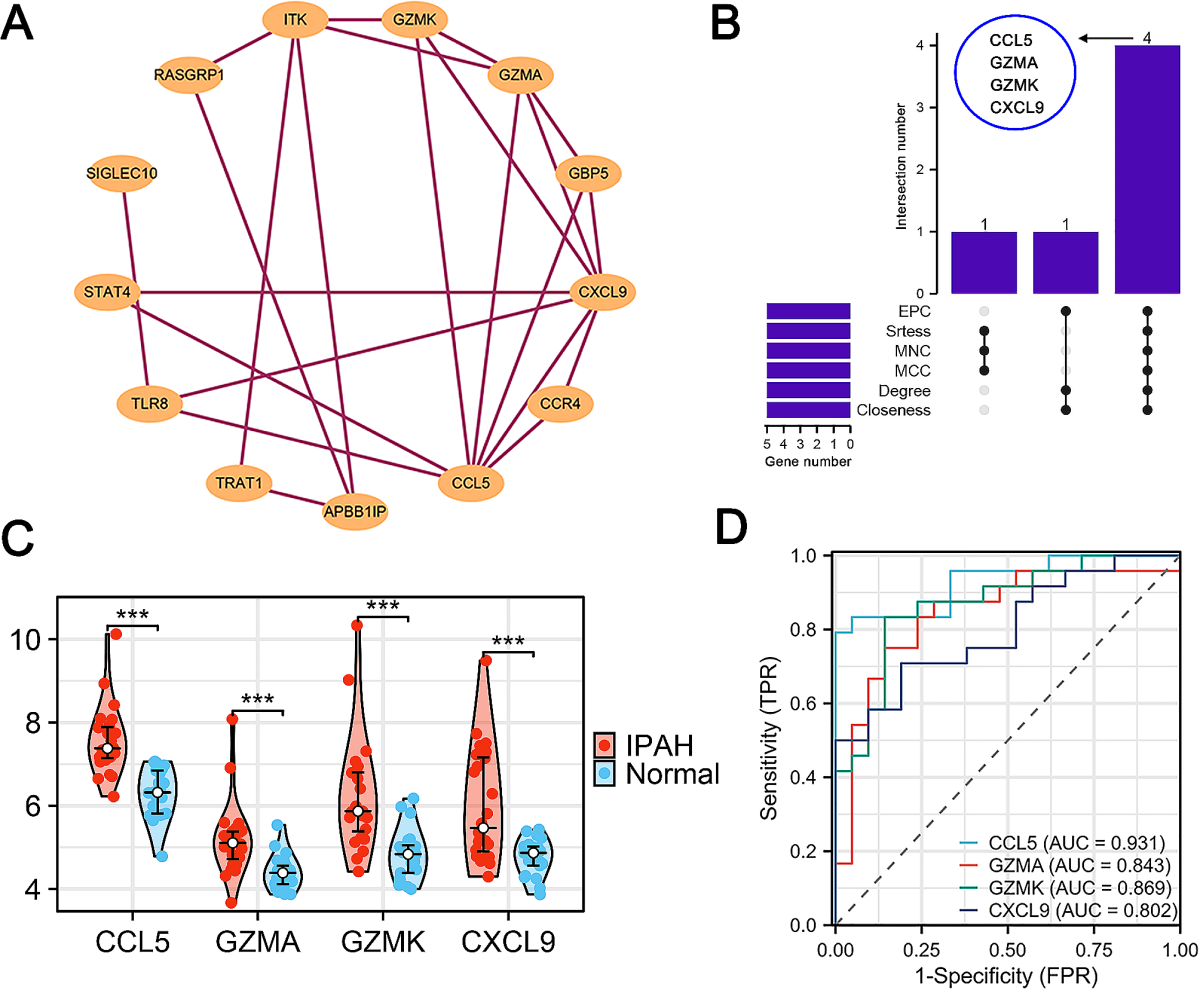
Characterization and diagnostic potential of inflammation-associated hub genes in idiopathic pulmonary arterial hypertension (IPAH). **A**. Protein-protein interaction network illustrating the interconnectivity between 22 inflammation-related genes, with lines indicating the interactions. **B**. UpSet plot depicting the intersection of hub genes identified by six distinct algorithms, with bar height representing the intersection size and connected dots indicating the combination of algorithms identifying the hub genes. **C.** Violin plots comparing the expression levels of the four hub genes (CCL5, GZMA, GZMK, CXCL9) between IPAH and the control group, with statistical significance denoted by asterisks. ***p < 0.001. **D.** Receiver operating characteristic (ROC) curves for the four hub genes, with the area under the curve (AUC) scores demonstrating their diagnostic performance in distinguishing between IPAH and control samples

In order to investigate the potential interaction of these 22 inflammation-related genes, a PPI analysis was conducted. The interaction network of these genes contained 13 nodes and 23 edges, as shown in Fig. 3A. The PPI network data were then imported into Cytoscape software to identify central hub genes. Hub genes were determined by intersecting the top five genes from six cytoHubba algorithms (Closeness, Degree, EPC, MCC, MNC, and Stress) (Additional file 6: Fig. S3C). As shown in Fig. 3B, genes encoding granzyme A (GZMA), granzyme K (GZMK), C-C motif chemokine ligand-5 (CCL5) and C-X-C motif chemokine ligand 9 (CXCL9) were identified as hub genes, which were significantly upregulated in IPAH compared to the controls (*p* < 0.05, Fig. 3C). Moreover, the ROC curve showed that these four hub genes had high AUC values and may serve as independent indicators for IPAH (Fig. 3D). Collectively, the interaction network revealed four central hub genes with potentially pivotal roles in the inflammatory response associated with IPAH.

### Hub gene expression in the MCT rat model

**Fig. 4:**
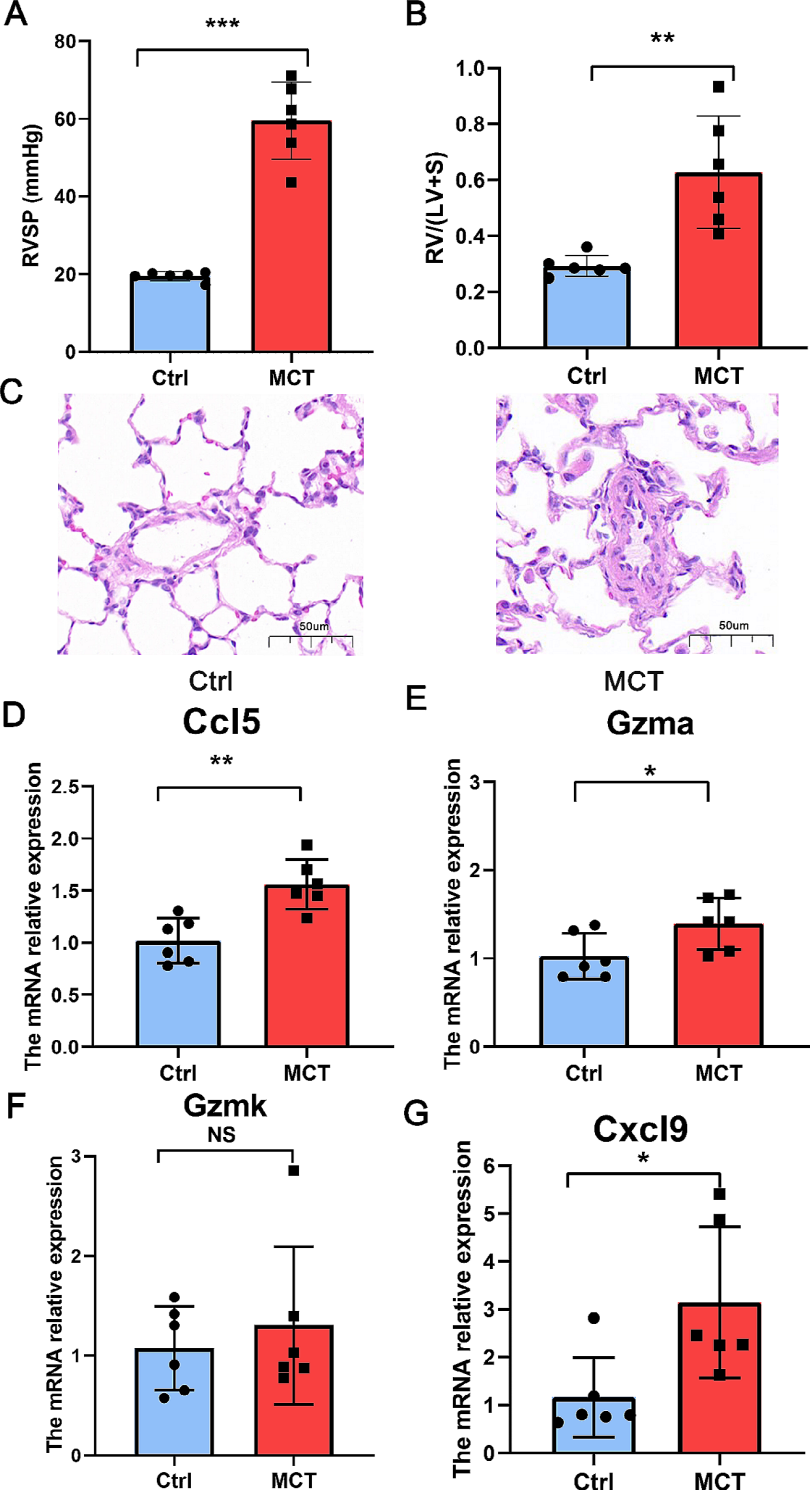
Expression of hub genes in the monocrotaline-induced rat model of pulmonary hypertension. **A. **Bar graph showing the right ventricular systolic pressure (RVSP), indicating a significant increase in the monocrotaline (MCT) group compared to controls (Ctrl) (***p < 0.001). **B.** Bar graph depicting the heart weight ratio (RV/(LV + S), right ventricle to the left ventricle plus septum), with the MCT group showing a significant increase (**p < 0.01). **C.** Hematoxylin and eosin (H&E) stained sections of the rat lung, revealing morphological changes of the pulmonary vasculature; scale bars represent 50 μm. **D-G.** Relative mRNA expression levels measured by RT-qPCR for the genes Gzma (**D**), Gzmk (**E**), Ccl5 (**F**), and Cxcl9 (**G**) between Ctrl and MCT groups, with statistical significance noted as *p < 0.05, **p < 0.01, NS: not significant

Subsequently, the expression of these four hub genes was investigated in the MCT-induced rat model of pulmonary hypertension. Compared to control animals, those in the MCT group exhibited pronounced pulmonary vascular remodeling (Fig. 4A). Additionally, the MCT group presented with significantly elevated right ventricular systolic pressure and (Fig. 4B) and right ventricular hypertrophy (Fig. 4C) (*p* < 0.05 for both), corroborating the successful induction of pulmonary hypertension. Analysis of lung tissue mRNA levels revealed a significant upregulation of Gzma, Ccl5, and Cxcl9 in the MCT group compared with the controls (*p* < 0.05 for all), while no significant difference in Gzmk expression was found between the two groups (*p* = 0.2293, Fig. 4D-G). Thus, GZMA, CCL5 and CXCL9 were considered as candidate genes for subsequent investigations of the exact cell types associated with their actions.

### Delineation of the cellular regulatory networks of the hub genes

**Fig. 5:**
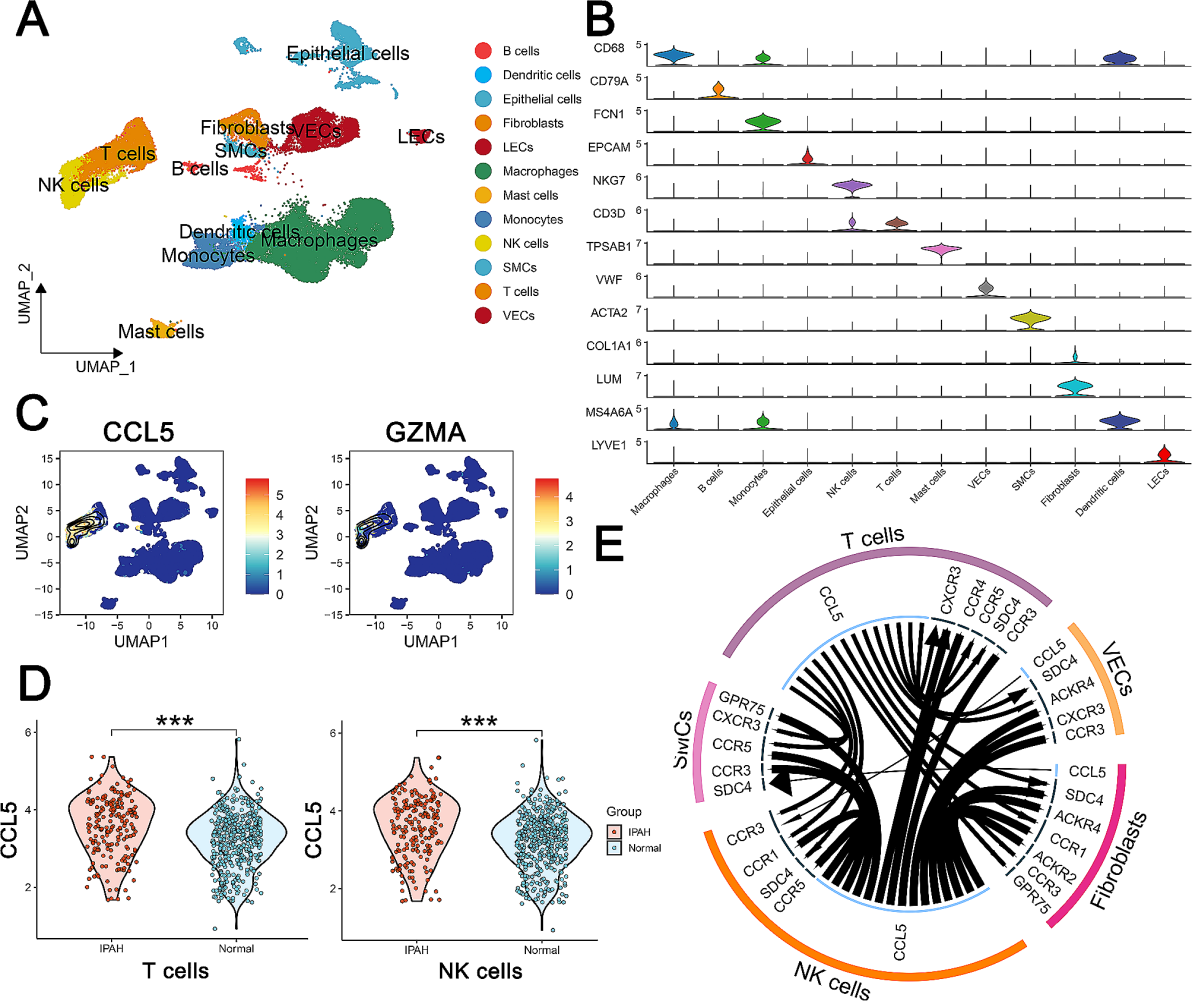
Single-cell RNA sequencing (scRNA-seq) analysis of cell-type-specific gene expression and intercellular interactions in idiopathic pulmonary arterial hypertension (IPAH). **A.** Uniform manifold approximation and projection (UMAP) visualization illustrating the diverse cell populations identified in normal and IPAH lung samples, with each cluster representing a unique cell type. **B.** Violin plots depicting the expression profiles of selected marker genes across the identified cell types, with each violin representing the distribution within a particular cell type **C.** UMAP plots showing the expression intensity of CCL5 and GZMA across all annotated cell types, with color intensity indicating expression levels. **D.** Violin plots contrasting the expression of CCL5 in T cells and natural killer (NK) cells between IPAH and normal samples, with significant differences highlighted (***p < 0.001). **E.** Ligand-receptor interaction circle plot detailing the potential communication pathways mediated by CCL5 among T cells, NK cells, vascular endothelial cells (VECs), smooth muscle cells (SMCs), and fibroblasts, suggesting a complex network of intercellular signaling in the IPAH lung tissue microenvironment. Statistical annotations: ***p < 0.001

ScRNA-seq data were employed to reveal primary source and target cell types for these three candidate genes. Following the exclusion of low-quality cells, 25 cell clusters were identified (Additional file 8: Fig. S4A). Using the top gene markers for each cluster (Additional file 8: Fig. S4B), the clusters were annotated into 12 cell types (Fig. 5A). The specific expression of marker genes by each cell type (Fig. 5B) confirmed the reliability of our cell type annotations. Notably, GZMA and CCL5 displayed high expression levels in T and natural killer (NK) cells (Fig. 5C), whereas the CXCL9 exhibited low expression across all cell types (Additional file 8: Fig. S4C), hence the subsequent analyses were centered on CCL5 and GZMA. We isolated T and NK cells exhibiting expression levels greater than zero for both CCL5 and GZMA, revealing a significant increase in CCL5 expression within these cell types in IPAH patients (Fig. 5D), whereas GZMA showed no difference between patient and control cells (Additional file 8: Fig.S4D). Importantly, the iTALK analysis revealed that CCL5 mediated the interaction of T and NK cells with vascular endothelial cells, vascular smooth muscle cells and fibroblasts through the same receptors, including CCR3 and SDC4 (Fig. 5E). while no interacting receptor was identified for GZMA. Taken together, the scRNA-seq results suggested that CCL5 is a crucial inflammatory factor secreted by T and NK cells, which is potentially implicated in vascular remodeling in IPAH owing to its regulatory effects on endothelial and smooth muscle cells.

## Discussion

In this study, we established a new analytical framework that integrated histopathology, microarray, and single-cell transcriptomic data to identify pivotal genes and immune cells implicated in IPAH. Using this methodology, we found that T cells and NK cells produced the inflammatory mediator CCL5, which interacted with pulmonary vascular cells to modulate their functions. Importantly, this innovative approach has elucidated a mechanism connecting vascular inflammation with pathological remodeling and pinpointed the critical factors involved.

The novelty of our investigation lies in the association between tissue histopathology and transcriptomic data. For histopathology, an inflammatory score was used to represent the extent of pulmonary vascular inflammation. This score quantifies perivascular infiltration by combining the abundance of inflammatory cells surrounding a vessel with the number of affected vessels, thereby substituting histological observations with a straightforward and accurate numerical value [2]. Our analysis showed a correlation between this score and key hemodynamic parameters, suggesting that the score not only reflected tissue histopathology, but was also relevant to functional indices of disease severity. Crucially, the usage of this score, which acted as an indicator of localized perivascular inflammation, provided an element of spatial characteristic to the transcriptomic data, thereby addressing a key limitation of microarray and scRNA-seq technologies [13]. In the analytical process, the inflammatory score was incorporated to guide the identification of important genes, while significant differences in gene expression were also taken into account. Additionally, the bioinformatic analysis examined the functions and interactions of these genes, narrowing down systematically to converge on a set of central hub genes. The expression levels of these hub genes could be used to distinguish IPAH patients from controls, and their altered expression levels were also verified in diseased rats, underscoring their significance. Furthermore, the exploitation of scRNA-seq analysis revealed the principal source and target cell types of these hub genes and their ligand-receptor interactions, providing a new mechanism underlying the phenomenon of vascular inflammation observed in IPAH [12].

The infiltration of various immune cells such as B cells, T cells, macrophages, and mast cells in the remodeled pulmonary arteries and around plexiform lesions is well-documented in IPAH patients [2, 23, 24]. Importantly, vascular inflammation has been suggested to significantly affect pulmonary hemodynamics [2]. Consistent with prior studies, we found a positive correlation between the pulmonary vascular inflammatory score with both mPAP and PVR. Furthermore, the genes associated with the inflammatory score were primarily involved in the regulation of T cell function. In particular, scRNA-seq data indicated that the key inflammatory factors were secreted mainly by T cells and NK cells. Our results thus revealed the identity of the genes connecting the immune cells in vascular inflammation and the altered hemodynamics observed clinically, highlighting T and NK cells as important immune cell types responsible for mediating inflammation in IPAH.

In terms of inflammatory factors, dysregulated cytokines and chemokines are commonly known to be involved in the pathogenesis of IPAH, modulating the function and proliferation of pulmonary vascular cells [8, 25, 26]. Among the extensive cytokine and chemokine families, our results specifically highlighted CCL5 as a key factor in the inflammatory response in IPAH, as determined through the integration of microarray analyses, scRNA-seq and animal modeling.

CCL5, also known as RANTES, is a CC chemokine family member known for its role as a pivotal chemotaxis inducer and its complex influence on various immune cell types [27]. Studies have demonstrated elevated CCL5 expression in the lung tissue and peripheral blood of pulmonary arterial hypertension (PAH) patients, which correlates with deteriorating cardiac function and adverse prognosis [28, 29]. It has been reported that CCL5 could be produced by endothelial cells in patients with PAH [28], where CCL5 deficiency increased apoptosis and tube formation of pulmonary artery endothelial cells (PAECs), and suppressed proliferation and migration of pulmonary artery smooth muscle cells (PASMCs) [30]. In addition, a recent study showed that CCL5 could also be secreted by macrophages, with the CCL5/CCR5 axis being a major molecular pathway mediating the regulation of macrophage-PASMC interactions, exerting a strong stimulatory effect on PASMC proliferation [8]. Intriguingly, our results revealed that CCL5 was mainly produced by T and NK cells and mediated the interaction of T and NK cells with vascular endothelial cells, smooth muscle cells, and fibroblasts through multiple molecular pathways. In support of our findings, CCL5 has been shown to be a key inducer of T cells [31], potentially resulting in their accumulation in the remodeled pulmonary vessels and initiating a vicious cycle in which more activated T cells escalate CCL5 production. Collectively, our findings reinforce the association between inflammatory mediators and pulmonary vascular remodeling, unveiling a novel molecular mechanism that involves CCL5 derived from T and NK cells. Further experimental studies are required to confirm this specific source of CCL5 and its downstream molecular targets in PAECs and PASMCs.

Given the newly discovered roles CCL5 and its receptor CCR3 in bridging T/NK cells and the altered functions of smooth muscle, endothelial, and fibroblast cells, this ligand-receptor pair may be considered as potential drug targets in IPAH. AKST4290, also known as BI144807 or ALK4290, is a highly specific small molecule antagonist of CCR3. Previous research has indicated that AKST4290 is safe in humans and showed efficacy in improving the vision of patients with age-related macular degeneration [32]. Future investigations could explore the potential effect of AKST4290 or similar inhibitors in the treatment of IPAH.

## Conclusions

In summary, using an innovative approach to integrate histopathological and transcriptomic data, we have performed comprehensive analyses to reveal that CCL5, as a key mediator produced by T and NK cells, was involved in the processes of vascular inflammation and remodeling in IPAH. This new analytical framework may accelerate translational research by transforming existing datasets into new and tractable targets for disease treatment.

Scatterplot of gene significance for the inflammatory score versus module membership.

Scatterplot correlating module membership with gene significance for inflammation, where the x-axis represents module membership in the inflammatory score-related module, and the y-axis denotes gene significance to inflammation (correlation coefficient and p-value are displayed).

### Electronic supplementary material

Below is the link to the electronic supplementary material.

Supplementary Material 1

Supplementary Material 2

Supplementary Material 3

Supplementary Material 4

Supplementary Material 5

Supplementary Material 6

Supplementary Material 7

Supplementary Material 8

Supplementary Material 9

## Data Availability

The datasets GSE117261 and GSE169471 used in the current study are available in the GEO repository (https://www.ncbi.nlm.nih.gov/geo/). The relevant coding scripts and key intermediate data of this study are available on GitHub (https://github.com/lixincheng888/Bulk-and-scRNA-analysis).

## Abbreviations

IPAH: Idiopathic pulmonary arterial hypertension
PAH: Pulmonary arterial hypertension
PH: Pulmonary hypertension
sc-RNA seq: Single cell RNA sequencing
GEO: Gene expression omnibus
WGCNA: Weighted gene co-expression network analysis
GO: Gene ontology
KEGG: Kyoto encyclopedia of genes and genomes
DEGs: Differentially expressed genes
PPI: Protein-protein interaction
ROC: Receiver operating characteristic
PAECs: Pulmonary artery endothelial cells
PASMCs: Pulmonary artery smooth muscle cells
mPAP: Mean pulmonary artery pressure
PVR: Pulmonary vascular resistance
MM: Module membership
GS: Gene significance
PCs: Principal components
RT-qPCR: Real-time quantitative PCR
UMAP: Uniform Manifold Approximation and Projection
GZMA: Granzyme A
GZMK: Granzyme K
CCL5: C-C Motif Chemokine Ligand-5
CXCL9: C-X-C Motif Chemokine Ligand 9
RVSP: Right ventricular systolic pressure
RVHI: Right ventricular hypertrophy index
RV/(LV + S): Weight ratio of the right ventricle to the left ventricle
