# Supplementary material for: A new integrative analysis of histopathology and single cell RNA-seq reveals the CCL5 mediated T and NK cell interaction with vascular cells in idiopathic pulmonary arterial hypertension
Source: J Transl Med. 2024 May 26;22:502. doi: 10.1186/s12967-024-05304-6 (PMC11129488; doi:10.1186/s12967-024-05304-6)
Supplement: Supplementary file 1 — Supplementary Material 1 [file 12967_2024_5304_MOESM1_ESM.docx]

**Table. S1 The information and metadata regarding the sequencing datasets.**

|  | GSE117261 | GSE169471 |
| --- | --- | --- |
| Sample Type | Lung | Lung |
| Data Type | Microarray | Single-cell RNA sequencing |
| Sequencing Platform | GPL6244 | GPL18573 and GPL20795 |
| Sample Number | 21 donor and  24 IPAH samples | 8 donor and  3 IPAH samples |

Abbreviations: IPAH: idiopathic pulmonary arterial hypertension.
