# Supplementary material for: A new integrative analysis of histopathology and single cell RNA-seq reveals the CCL5 mediated T and NK cell interaction with vascular cells in idiopathic pulmonary arterial hypertension
Source: J Transl Med. 2024 May 26;22:502. doi: 10.1186/s12967-024-05304-6 (PMC11129488; doi:10.1186/s12967-024-05304-6)
Supplement: Supplementary file 2 — Supplementary Material 2 [file 12967_2024_5304_MOESM2_ESM.docx]

**Table S2. PCR primers of genes**

| Gene | Primer sequence (5' to 3') |
| --- | --- |
| Gzma | Forward: TTTCCGAGGCATCACAGCTT |
|  | Reverse: CCTTTAAACAGCGCCCTTCG |
| Gzmk | Forward: GAGTCCTGATCCATCCGCAG |
|  | Reverse: CCGTGCGAAGCTTTATCAGC |
| Ccl5 | Forward: GTGCCCACGTGAAGGAGTA |
|  | Reverse: TCTTCTCTGGGTTGGCACAC |
| Cxcl9 | Forward: TGTGGAGTTCGAGGAACCCT |
|  | Reverse: ACCCTTGCTGAATCTGGGTC |
| β-Actin | Forward: GAGAGGGAAATCGTGCGTGA |
|  | Reverse: TGGAAGGTGGACAGTGAGGC |
