# Supplementary figures and images for: A new integrative analysis of histopathology and single cell RNA-seq reveals the CCL5 mediated T and NK cell interaction with vascular cells in idiopathic pulmonary arterial hypertension
Source: J Transl Med. 2024 May 26;22:502. doi: 10.1186/s12967-024-05304-6 (PMC11129488; doi:10.1186/s12967-024-05304-6)

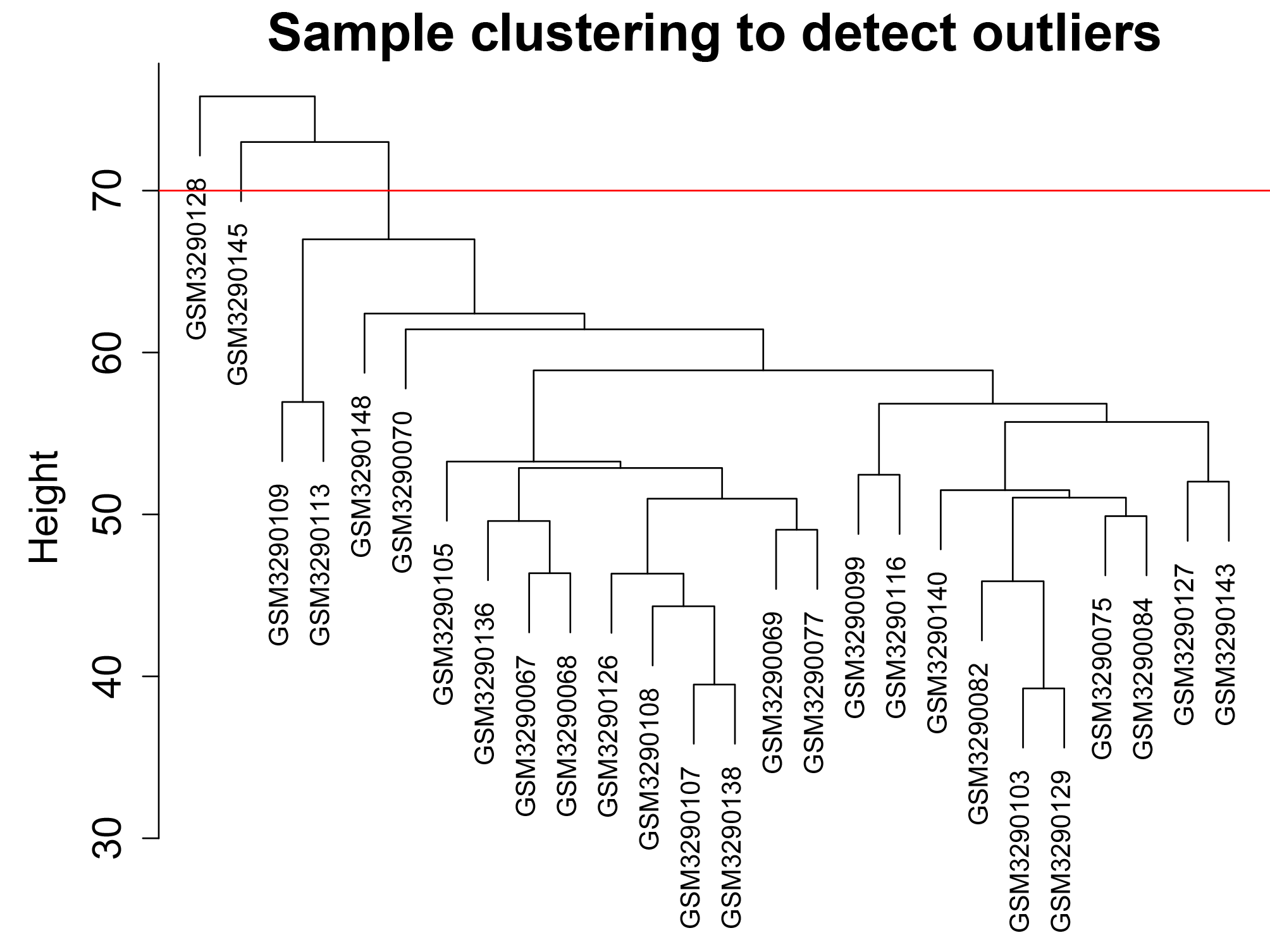

Supplement: Supplementary file 3 — Supplementary Material 3 [file 12967_2024_5304_MOESM3_ESM.tif]

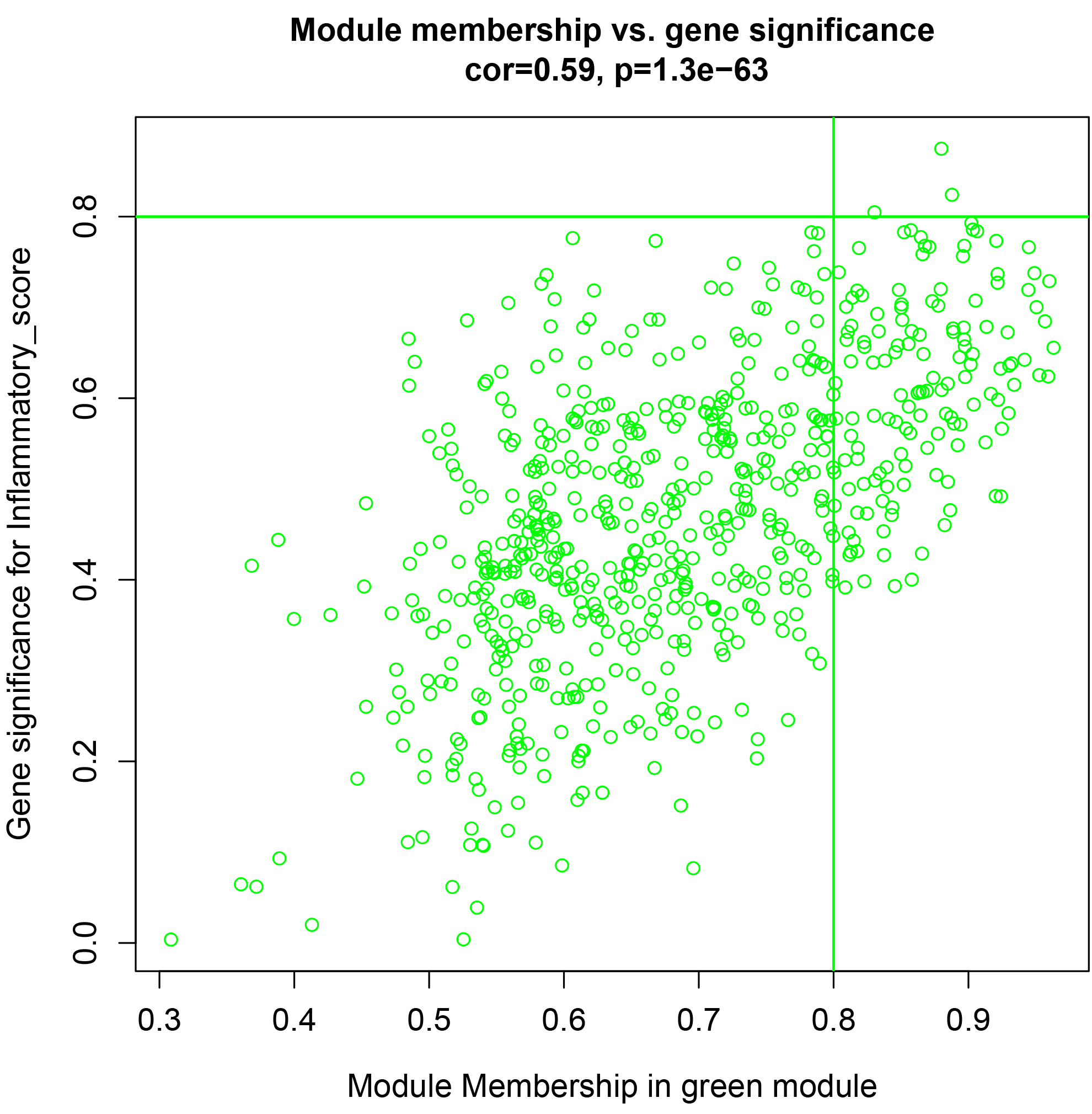

Supplement: Supplementary file 5 — Supplementary Material 5 [file 12967_2024_5304_MOESM5_ESM.tif]

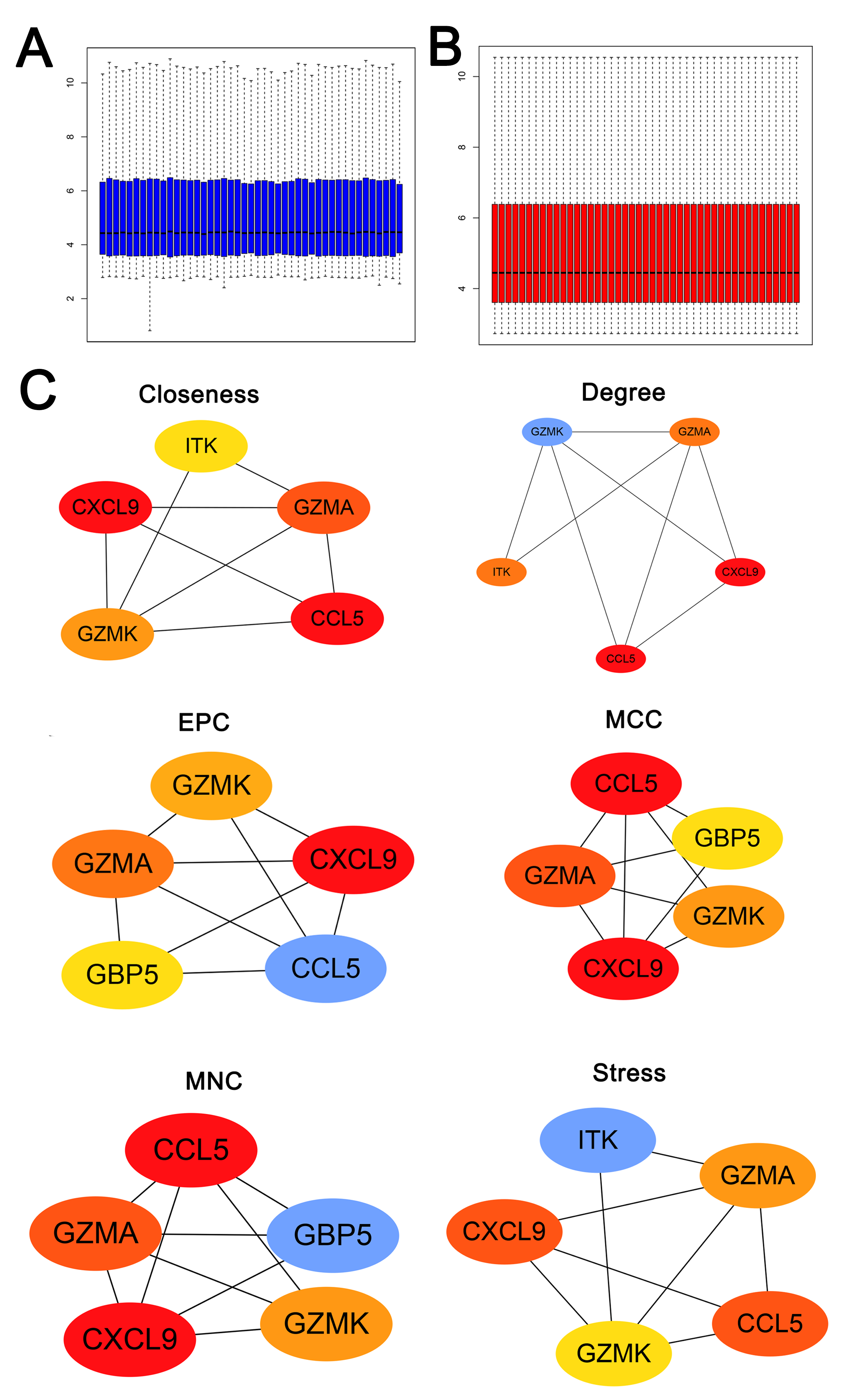

Supplement: Supplementary file 6 — Supplementary Material 6 [file 12967_2024_5304_MOESM6_ESM.tif]

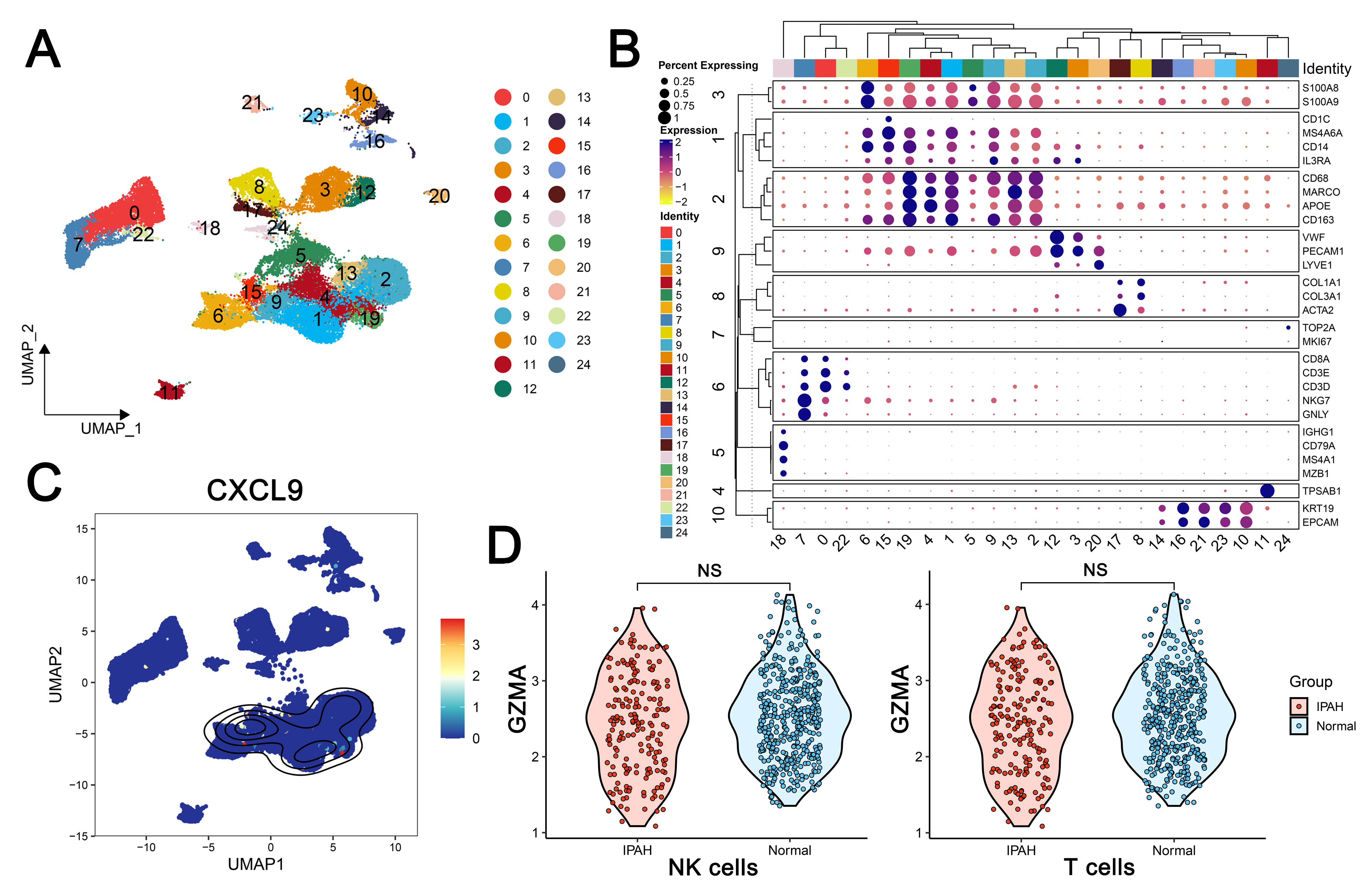

Supplement: Supplementary file 8 — Supplementary Material 8 [file 12967_2024_5304_MOESM8_ESM.tif]
