# Supplementary material for: A new integrative analysis of histopathology and single cell RNA-seq reveals the CCL5 mediated T and NK cell interaction with vascular cells in idiopathic pulmonary arterial hypertension
Source: J Transl Med. 2024 May 26;22:502. doi: 10.1186/s12967-024-05304-6 (PMC11129488; doi:10.1186/s12967-024-05304-6)
Supplement: Supplementary file 4 — Supplementary Material 4 [file 12967_2024_5304_MOESM4_ESM.docx]

**Table S3. Demographics of patients with idiopathic pulmonary arterial hypertension**

|  | IPAH (n=24) |
| --- | --- |
| Age (years) | 45.92 ± 2.77 |
| Female | 18 (75.0%) |
| Pulmonary hemodynamics |  |
| mPAP (mmHg) | 50.00 (47.00-59.25) |
| PVR (Wood Units) | 10.53 ± 1.10 |
| Histopathological index |  |
| Inflammatory score | 0.60 (0.26-0.74) |
| Adventitia thickness | 0.17 ± 0.01 |
| Media thickness | 0.14 ± 0.01 |
| Intima thickness | 0.33 ± 0.25 |
| Total thickness | 0.81 ± 0.30 |

Abbreviations: IPAH: idiopathic pulmonary arterial hypertension; mPAP: mean pulmonary artery pressure; PVR: pulmonary vascular resistance.
