# Supplementary material for: A new integrative analysis of histopathology and single cell RNA-seq reveals the CCL5 mediated T and NK cell interaction with vascular cells in idiopathic pulmonary arterial hypertension
Source: J Transl Med. 2024 May 26;22:502. doi: 10.1186/s12967-024-05304-6 (PMC11129488; doi:10.1186/s12967-024-05304-6)
Supplement: Supplementary file 9 — Supplementary Material 9 [file 12967_2024_5304_MOESM9_ESM.docx]

**Supplementary Material Information**

**Additional file 1: Table S1.**

The information and metadata regarding the sequencing datasets.

**Additional file 2:** **Table S2.**

Sequences of qPCR primers used in this study.

**Additional file 3: Fig. S1.**

Sample clustering to detect outliers.

The hierarchical clustering dendrogram displays sample clustering to detect outliers, with the height indicating the distance between clusters. Outlier samples are marked with a red horizontal line.

**Additional file 4, Table S3.**

Demographics of patients with idiopathic pulmonary arterial hypertension.

**Additional file 5: Fig. S2.**

Scatterplot of gene significance for the inflammatory score versus module membership.

Scatterplot correlating module membership with gene significance for inflammation, where the x-axis represents module membership in the inflammatory score-related module, and the y-axis denotes gene significance to inflammation (correlation coefficient and p-value are displayed).

**Additional file 6: Fig. S3.**

Normalized analysis of gene expression and hub gene identification.

**A**. Bar plot illustrating the gene distribution of each sample before normalization. **B**. Bar plot illustrating the gene distribution of each sample after normalization. **C.** The top 5 hub genes identified by the six algorithms (closeness, degree, EPC, MCC, MNC, and stress).

**Additional file 7: Table S4.**

Differentially expressed genes between IPAH and normal samples.

**Additional file 8: Fig. S4.**

Single-cell RNA sequencing data revealing cellular heterogeneity and gene expression in immune cells.

**A.** Uniform manifold approximation and projection (UMAP) plot categorizing cells into clusters (0-24), each denoted by a unique color. **B.** Heatmap showing the expression levels of selected genes across the identified clusters. The size of the dots represents the percentage of cells expressing the gene within each cluster, while the color intensity reflects the expression level. **C.** UMAP plot showing the expression of CXCL9 across all cells, with color intensity indicating expression level and contour lines demarcating cell density regions. **D.** Violin plots comparing the expression of GZMA in NK and T cells between IPAH and normal controls. NS: not significant.
